# Disrupting cell death: ferroptosis and pyroptosis inhibition in hepatic ischemia-reperfusion injury via modulation of GPX4 and cGAS pathways by deferoxamine and octreotide

**DOI:** 10.3389/fphar.2025.1610718

**Published:** 2025-12-09

**Authors:** Rasha A. Tawfiq, Yasmeen M. Attia, Hameis M. Sleem, Mai El-Sayed Ghoneim

**Affiliations:** 1 Department of Pharmacology, Faculty of Pharmacy, The British University in Egypt, Cairo, Egypt; 2 Drug Research and Development Group (DRD-G), Health Research Center of Excellence, The British University in Egypt, Cairo, Egypt; 3 Department of Biochemistry, Faculty of Pharmacy, The British University in Egypt, Cairo, Egypt; 4 Department of Pharmacology and Toxicology, Faculty of Pharmacy, University of Sadat City (USC), Sadat City, Egypt

**Keywords:** liver, ferroptosis, pyroptosis, octreotide, deferoxamine, ischemia-reperfusion

## Abstract

Hepatic ischemia-reperfusion injury (HIRI) is a significant complication of liver transplantation, often precipitating postoperative liver dysfunction/failure. Given the rising global prevalence of end-stage liver disease, which necessitates liver transplantation, protecting against HIRI is crucial. Here, we investigated the effects of octreotide (OCT), a pyroptosis inhibitor, and deferoxamine (DEF), a ferroptosis suppressor, individually and combined in the HIRI model. Their impact on cyclic GMP-AMP synthase/stimulator-of-interferon (cGAS/STING) genes and glutathione peroxidase 4 (GPX4) activity was explored. Male rats (n = 4–5) were subjected to 30 min ischemia followed by 3 h reperfusion and treated with OCT (75 μg/kg; 30 μg/kg, i.p. & 45 μg/kg, s.c.), DEF (200 mg/kg, i.p.), or their combination 30 min prior to ischemia. DEF and the combined therapy alleviated HIRI histopathologically compared to OCT, while all treatments improved liver function. Ferroptosis suppression was noticeable with DEF and the combined regimen, likely via GPX4 activation and cyclooxygenase 2 (COX2) inhibition. All treatments displayed anti-inflammatory activity through suppressing toll-like receptor 4 (TLR4)/nuclear factor-kappa-B (NF_k_B) axis and pyroptosis. The combination, however, lacked anti-pyroptotic activity. These alterations paralleled cGAS downregulation, independent of STING modulation. Collectively, DEF conferred superior hepatoprotection compared to OCT, primarily due to its antioxidant and anti-inflammatory activities. Combinatorial therapy amplified the modulation of GPX4, COX2, and TLR4/NFκB without additive antipyroptotic activity.

## Introduction

Chronic liver diseases are often silent, yet they can lead to end-stage liver disease (ESLD), which is a mounting cause of mortality globally. This global high prevalence of ESLD raises the need for liver transplantation since it is reported as the best curative option for those patients (Liu and Man, 2021). One of the main reasons behind the high probability of liver transplantation failure is hepatic ischemia-reperfusion injury (HIRI) (Liu and Man, 2023). Generally, ischemia-reperfusion (IR) is a pathological condition that happens when the blood circulation to an organ is restrained (ischemia), followed by blood flow restoration and re-oxygenation (reperfusion). During ischemia, tissue hypoxia may occur due to the imbalance between metabolic supply and demand. However, upon organ reperfusion, innate and adaptive immune responses arise. Furthermore, exacerbated inflammatory reactions and programmed cell death, like necrosis, apoptosis, and autophagy, are activated (collectively named as “reperfusion injury”). IR injury may occur not only following organ transplantation but also in several other conditions, like cardiac arrest, obstructive sleep apnea, hepatic resection surgery, states of trauma or shock, and episodes of oxygen deprivation in sickle cell anemia. IR injury in one organ could affect other organs remotely through potentiating inflammation in different organs, leading to multiple organ failure (Brakenridge et al., 2024). Thus, HIRI, for instance, may lead to inflammatory responses in the intestine, kidneys, pancreas, lungs, or adrenal glands, which can cause multiorgan dysfunction syndrome (Rampes and Ma, 2019). Concisely, IR inflammation may lead to short-and long-term outcomes. The short-term effects include the likelihood of acute rejection and causing liver graft malfunction or non-function. However, the long-term outcome could lead to fibrosis, cancer recurrence, chronic organ rejection, or impaired regeneration (Liu and Man, 2021). Consequent to these HIRI complications, the high probability of donor mortality, the incidence of transplant rejection, altogether, affect the perspective of organ donation negatively (Rampes and Ma, 2019). Therefore, it is crucial to prevent HIRI to save the lives of donors and recipients of the liver in the case of transplantation (Liu and Man, 2021).

Recently, it has been reported that ferroptosis, one of the regulated cell death programs, is linked to ischemia-reperfusion (IR). Ferroptosis is an oxidative, iron-dependent form of cell death in which iron overload causes an elevation in reactive oxygen species (ROS) levels and lipid peroxidation, leading to oxidative mitochondrial damage and organ dysfunction during IR. This mechanism could be counteracted by using either ferroptosis inhibitors such as the ferroptosis-specific inhibitor ferrostatin-1 (Fer-1) and liproxstatins-1, or iron chelators like deferoxamine (DEF) (Yamada et al., 2020; Khan et al., 2025). Ferroptosis has recently been introduced as a form of autophagy (Luo et al., 2021) and is referred to as “ferritinophagy” (Li W. et al., 2021).

Another type of programmed cell death that may be involved in IR is pyroptosis. It is a highly inflammatory non-apoptotic cell death mediated by NOD-, LRR- and pyrin domain-containing protein 3 (NLRP3) inflammasome and cysteinyl aspartate specific proteinase-1 (Caspase-1). Notably, NLRP3 inflammasome comprises 3 domains, NLRP3 receptor or sensor, an adaptor domain called apoptosis-associated speck-like protein containing a CARD (ASC), and the effector pro-caspase-1. Furthermore, NIMA-related kinase 7 (NEK7) oligomerizes with NLRP3 into a complex essential for ASC formation and caspase-1 activation. On activation of NLRP3 inflammasome, pro-caspase-1 is converted into cleaved/active caspase-1. The latter initiates the maturation of the precursor cytokines pro-interleukin (IL)-1β and pro-IL-18 into their biologically active forms, IL-1β and IL-18. The active forms of interleukins cause the cleavage of gasdermin D (GSDMD) into its N terminal form, creating pores in the cell membrane and leading to the release of cell contents extracellularly (Swanson et al., 2019; Liu and Man, 2021).

Many strategies have been used to prevent HIRI, like counteracting oxidative stress and inflammation via antioxidants and immunomodulatory therapies such as melatonin, caspase inhibitors, selectin inhibitors, protein kinase B (Akt) activators, AMPK activators, PPARγ agonists, and miRNA-based therapy (Rampes and Ma, 2019). Meanwhile, there is no optimum therapeutic regimen for managing HIRI so far (Mu et al., 2022).

Somatostatin, a multifactorial neuropeptide growth hormone inhibitor, is produced by immune and neuroendocrine cells in response to neuropeptides, nutrients, ions, cytokines, growth factors, steroids, and thyroid hormones (Gomaa et al., 2020). Somatostatin has been demonstrated to decrease the production of ROS by leukocytes, influence leukocyte infiltration, adhesion, and chemotaxis (Wang et al., 2015). Octreotide (OCT), a potent synthetic long-acting somatostatin analog, has been used as a treatment for many diseases, including gastrointestinal disorders and hypoglycemia (Xu et al., 2017; Galvin and Wong, 2019). It is reported that OCT can protect against IR damage in several organs like retina (Wang et al., 2015), pancreas (Woeste et al., 2008), ovary (Yildirim et al., 2015), intestine (Takano et al., 2012), kidney (Xu et al., 2017), heart (Gomaa et al., 2020), and liver (Yang et al., 2013; Zou et al., 2018; Mohamed et al., 2021). One of the recently reported mechanisms that mediate the action of OCT in preventing HIRI is the inhibition of pyroptosis through suppressing toll-like receptor 4 (TLR4)/nuclear factor-kappa-B (NF_k_B)/NLRP3 axis (El-Sisi et al., 2021). Other reports highlighted the effect of OCT as an autophagy inducer in preventing HIRI through the activation of the Keap1/Nrf2 system (Zou et al., 2018; Mohamed et al., 2021).

Due to its iron chelating property, DEF (also known as desferal or desferrioxamine) has been reported to help in preventing the IR in some animal models like IR following peripheral nerve compression (Li et al., 1996), cardiac IR by chelating iron, which is responsible for the production of ROS (Ramesh Reddy et al., 1989), or through promoting HIF-1/BNIP3-mediated mitochondrial autophagy (Yang et al., 2020) and in HIRI (Polat et al., 2006; Arkadopoulos et al., 2010).

The cyclic GMP-AMP synthase (cGAS)/stimulator-of-interferon genes 1(STING1) pathway has emerged as a critical mediator of innate immune responses, particularly in sensing cytosolic DNA, which can be released during cellular stress or damage, such as that occurring during IR injury (Wang et al., 2025; Zhu et al., 2025). Activation of cGAS/STING1 can lead to the production of type I interferons and other pro-inflammatory cytokines, exacerbating inflammation and contributing to cell death pathways, including pyroptosis and potentially ferroptosis (Ding et al., 2024; Liu et al., 2024). Given its central role in inflammatory signaling and cross-talk with various cell death mechanisms, targeting the cGAS/STING1 pathway represents a promising therapeutic axis to mitigate HIRI-associated inflammation and cell death. Therefore, investigating the implication of cGAS/STING1 in the mechanism of action of HIRI interventions is crucial for a comprehensive understanding.

From the previously reported data, it was hypothesized that by combining a pyroptosis inhibitor, like OCT, with a ferroptosis inhibitor, like DEF, a promising effect may be shown in the rodent HIRI model. Thus, this study aimed to study, for the first time to our knowledge, the efficacy of combining OCT with DEF in preventing HIRI damage and investigate the mechanistic insights of this new combination.

## Materials and methods

### Drugs and chemicals

Octreotide was obtained from TASH Biotechnology (Shanghai, China), deferoxamine was obtained from Novartis (Cairo, Egypt) and other chemicals were of analytical grade.

### Animals

Twenty-four adult male Sprague Dawley rats, weighing 250–280 g (10–12 weeks old), were purchased from the animal house of the Faculty of Pharmacy, British University in Egypt (BUE), Egypt. The rats were placed in standard cages (2–3 rats per cage) under suitable laboratory conditions with ambient temperature (22 ± 3 °C), relative humidity (50%–70%), and a 12-h light-dark cycle. They were supplied with commercial food and permitted to drink water *ad libitum*. The rats were acclimatized for a week at the animal facility. The experimental procedures comply with the Guide for the Care and Use of Laboratory Animals (National Research Council, 2011) and ARRIVE guidelines. The study protocol was approved by the research ethics committee of the Faculty of Pharmacy, British University in Egypt (BUE) (approval no. EX2406).

### Induction of surgical technique

All rats were subjected to anesthesia using a mixture of 100 mg/kg ketamine and 10 mg/kg xylazine (Atef et al., 2017) after fasting overnight. An abdominal midline was done after shaving and adding 70% ethanol for disinfection. After that, the rats were subjected to 70% partial ischemia by clamping the hepatic pedicle using a microvascular bulldog clamp for 30 min (Xue et al., 2016). During this period, the abdomen area was covered with wet gauze to reduce the risk of dehydration, and the rats were kept warm by being placed on a heated pad to regulate their body temperature. Subsequently, the clamp was removed to facilitate reperfusion for a duration of 3 h (Xue et al., 2016). Subsequently, the abdominal muscles and skin were meticulously sutured in distinct layers. It is important to mention that the rats in the sham group underwent a laparotomy procedure without any clamping.

### Experimental design

Rats were randomly allocated into five groups (n = 4–5 rats per group); the 1^st^ group (sham) was subjected to laparotomy and received the vehicles. The 2^nd^ group (HIRI) was subjected to hepatic IR injury (30 min/3 h). The 3^rd^ group (HIRI + OCT) was subjected to HIRI and received 75 μg/kg of OCT (30 μg/kg, i.p., and 45 μg/kg, s.c.) 30 min before the onset of ischemia (El-Sisi et al., 2021). The 4^th^ group (HIRI + DEF) was subjected to HIRI and received DEF (200 mg/kg, i.p.) 30 min before the onset of ischemia (Yang et al., 2020). The 5^th^ group (HIRI + OCT/DEF) was subjected to HIRI and received OCT (75 μg/kg) and DEF (200 mg/kg) according to the above-mentioned treatment protocol. Group sizes (n = 4–5) were not equal across all experimental groups, as they were determined in accordance with the principle of “Reduction” in the 3Rs, while ensuring sufficient power to detect biologically relevant differences based on prior study (XUE et al., 2016).

### Sample collection

At the end of reperfusion, the rats were euthanized while under the influence of halothane anesthesia. Initially, blood samples were collected, centrifuged at 3,000 rpm for sera separation, and then stored at −20 °C. After that, the liver samples were split into two portions; one portion was preserved in 10% phosphate-buffered formalin for histopathological and immunohistochemical examinations, while the other one was aliquoted and kept at −80 °C for biochemical assessments and real-time quantitative polymerase chain reactions (qRT-PCR).

### Liver enzymes assessment

The liver function tests were assessed by measuring serum alanine aminotransferase (ALT; Cat no. E.C.2.6.1.2.) using commercial kits provided by Spectrum Diagnostic Company, Cairo, Egypt.

### Oxidative stress assessment

The hepatic oxidative stress was determined by measuring the levels of malondialdehyde (MDA; Cat. No. MD2529), superoxide dismutase (SOD; Cat, No. SD2521), and catalase (CAT; Cat. No. CA2517; Elabscience®, United States) using commercial kits provided by Bio-Diagnostic Company (Giza, Egypt).

### Ferroptotic markers assessment

The concentration of total iron binding capacity (TIBC) was determined using commercial kits (Cat. No. TI1511), provided by Bio-Diagnostic Company (Giza, Egypt). Furthermore, the concentration of iron in tissue was determined by Inductively Coupled Plasma Optical Emission Spectrometer (ICP-OES) (Agilent 5100 Synchronous Vertical Dual View (SVDV), MY15180008) following tissue digestion with nitric acid. Standard iron solution used (Cat. No. HC15460381) was purchased from Merck KGaA (Darmstadt, Germany) to assess the Fe concentration in tissue samples in mg iron/kg tissue according to the standard method for examination of (Lipps et al., 2023). Additionally, glutathione peroxidase activity (GPX4; Cat no. E-BC-K883-M) was determined in liver homogenate samples using a commercial colorimetric kit purchased from Elabscience®, United States.

### Inflammatory markers assessment

The concentration of toll-like receptor-4 (TLR4) and phosphorylated nuclear factor kappa B (NFĸB-p65) were estimated using Rat TLR4 ELISA Kit (Cat no. E-EL-R0990, Elabscience®, United States), and Rat NFĸB-p65 ELISA Kit (Cat no. BYEK3040, United States Chongqing Biospes Co., Ltd, China), respectively, in the liver homogenate samples following the commercial ELISA kits manufacturer’s instructions. The protein levels of TLR4 and NFĸB-p65 obtained were normalized to their respective protein content in each sample, which was assessed chemically according to the Lowry method (Lowry et al., 1951).

### Histopathological assessment

For histopathological assessment, liver tissue specimens were embedded in paraffin wax, sliced into 5 µm sections, and stained with hematoxylin and eosin (H&E) (Bancroft and Gamble, 2008). After that, the slices were examined under a light microscope for evaluation of liver injury by a pathologist who was unaware of the grouping of the rats.

### Immunohistochemical examination

Liver tissue sections were prepared on adhesive slides, deparaffinized, and rehydrated in distilled water. Subsequently, a heat-induced epitope retrieval step was performed, followed by incubation with primary anti-cyclooxygenase 2 (COX2) at a dilution of 1:200 (Cat no. sc-19999, Santa Cruz Biotechnology, Inc., United States) for 1 hour at room temperature. Following washing, the HRP-labelled detection kit (BioSB, United States) was employed according to the manufacturer’s recommendations to produce the color. Mayer’s hematoxylin is used as a counterstain. Negative control slides were generated by omitting incubation with primary antibodies. The positive expression was assessed as a percentage of area stained in nine systematic, randomly selected microscopic fields for each group using Olympus cellSens Dimension imaging software. The examination was done by a pathologist who was blind to the grouping of the rats.

### RNA extraction and cDNA synthesis

Total RNA was isolated from approximately 30 mg of rat liver tissue using RNeasy Minikit (Cat. no. 174104, Qiagen, MA, United States) following the manufacturer’s protocol. Tissue homogenization was performed using a rotor-stator homogenizer to ensure efficient cell disruption and RNA release. Following RNA isolation, both RNA concentration and purity were quantified by determining the A260/A280 ratio using a Q5000 UV-Vis Nanodrop spectrophotometer (Quawell Technology, CA, United States). Subsequently, RNA was reverse transcribed into complementary DNA (cDNA) using the GoScript Reverse Transcription Kit (Cat. No. A5000, Promega) according to the manufacturer’s instructions. This step generates a more stable DNA molecule suitable for downstream gene expression analyses.

### Quantitative real-time PCR (qRT-PCR)

Gene expression levels of NOD-, LRR- and pyrin domain-containing protein 3 (NLRP3) receptor or sensor, the adaptor domain an apoptosis-associated speck-like protein containing a CARD (ASC), caspase-1, NIMA-related kinase 7 (NEK7), interleukin (IL)-1β, IL-18 and Gasdermin D (GSDMD)-N terminal, and cyclic GMP-AMP synthase (cGAS)/stimulator-of-interferon genes 1 (STING1) were quantified using quantitative real-time PCR (qRT-PCR) on a StepOnePlus Real-Time PCR System (Applied Biosystems, CA, United States). The reaction mixture, with a final volume of 20 μL, consisted of 10 µL GoTaq® qPCR Master Mix (Cat. No. A6001, Promega), 0.4 µM of each forward and reverse primer, and 2 µL of diluted cDNA. Primers were obtained from Promega. Relative gene expression was determined using 2^-ΔΔCT ±standard error of mean (SEM). A complete list of primer sequences is provided in Table 1. The examination procedures were done by a blind investigator.

**TABLE 1:** Primer sequences used in qRT-PCR.

No.	Primer	Primer sequence
1	ASC-forward	GTT​GAT​GGT​TTG​CTG​GAT​GC
	ASC-reverse	AGT​TCT​TGC​AGG​TCA​GGT​TC
2	Caspase1- forward	ACA​AGA​TCC​TGA​GGG​CAA​AG
	Caspase1- reverse	GTA​TCC​ATC​TCT​TCC​TGG​TTC​AG
3	IL1β - forward	GCTTCCAAGCCCTTGACT
	IL1β - reverse	AAT​GAG​TGA​CAC​TGC​CTT​CC
4	IL18- forward	CAA​CGA​ATC​CCA​GAC​CAG​AC
	IL18- reverse	TTA​CAG​GAG​AGG​GTA​GAC​ATC​C
5	cGAS- forward	GCT​AAA​GAA​GGT​GCT​GGA​CAA
	cGAS- reverse	ACT​CCC​GTC​TCT​GCA​TTC​T
6	GSDMD- forward	TGCAACAGCTGCGGAAT
	GSDMD- reverse	TGTGGACCTGGGTGATCT
7	NEK7- forward	ACG​ACC​AGA​TAT​GGG​CTA​T
	NEK7- reverse	GAA​CTC​CAT​CCA​GGA​GAC​A
8	NLRP3- forward	CTT​CAG​GCT​GAT​CCA​AGA​GAA​T
	NLRP3- reverse	CAG​TCT​CCA​TCT​GCT​GCT​TTA
9	STING1- forward	GGC​TGT​ATA​TCC​TCT​TCC​CAT​TG
	STING1- reverse	GGG​CAG​CAT​ATC​TCG​GAA​TC
10	GAPDH- forward	CCT​CGT​CTC​ATA​GAC​AAG​ATG​GT
	GAPDH- reverse	GGG​TAG​AGT​CAT​ACT​GGA​ACA​TG

NLRP3: NOD-, LRR- and, pyrin domain-containing protein 3 receptor, ASC: apoptosis-associated speck-like protein containing a CARD, NEK7: NIMA-related kinase 7, IL-1β: interleukin-1β, IL-18: interleukin-18, and GSDMD: Gasdermin D, cGAS: cyclic GMP-AMP, synthase, STING1: stimulator-of-interferon gene-1.

### Statistical analysis

The results were expressed as mean ± SEM. Normality testing was performed using the Brown-Forsythe test, and the outliers were checked by Grubbs’ test. The statistical differences between groups were analyzed using one-way analysis of variance (ANOVA), followed by *Tukey’s post hoc* test for multiple comparisons between groups. The statistical significance was determined at p < 0.05. All statistical tests and graphical presentations were conducted using GraphPad Prism version 9.0 (GraphPad Prism Software, CA, United States).

## Results

### Effect of OCT and/or DEF on histopathological changes against hepatic ischemia/reperfusion injury in rats

**FIGURE 1:**
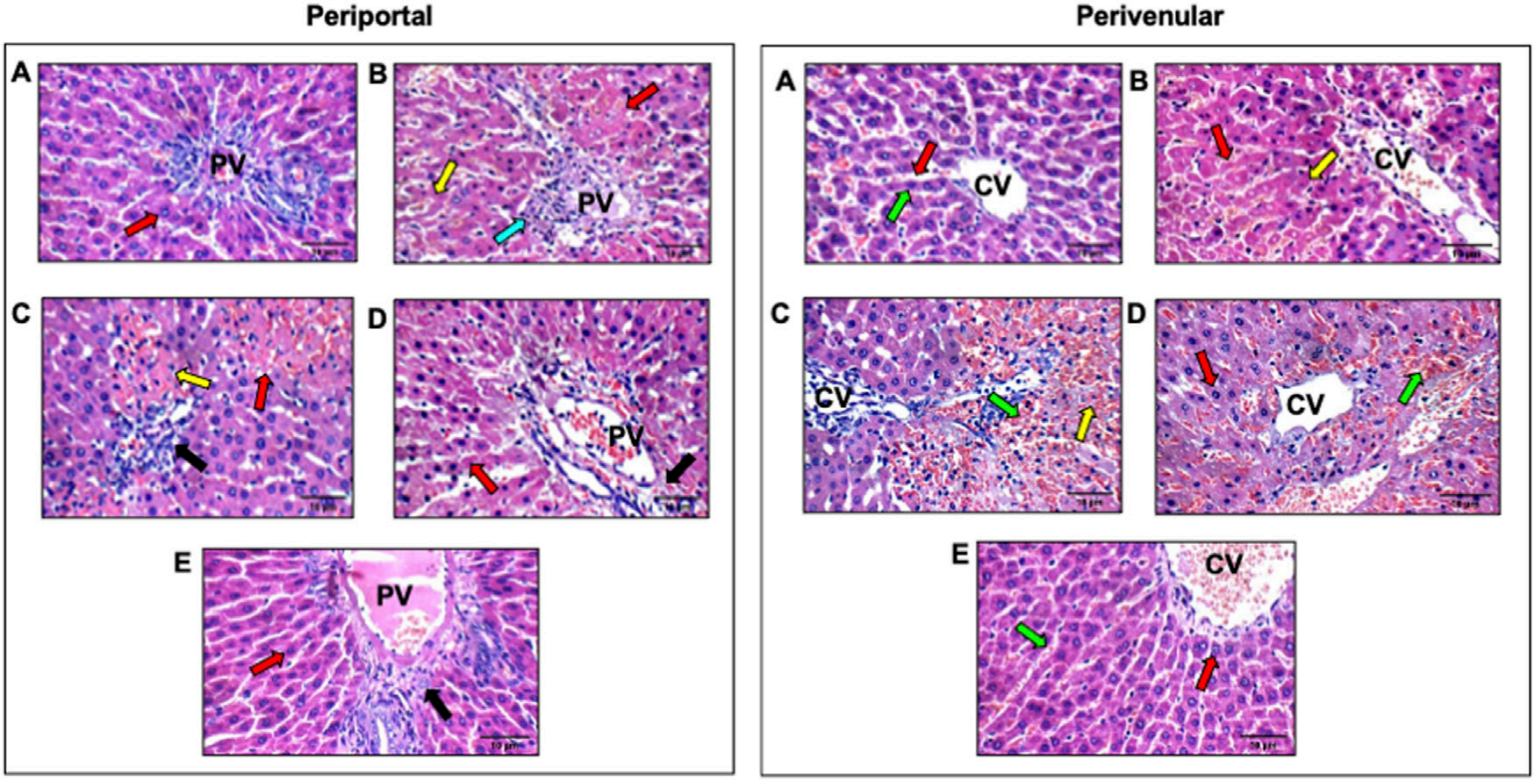
Effect of OCT and/or DEF pretreatment on histopathological changes against hepatic ischemia/reperfusion injury in periportal and perivenular zones of liver in rats (H&E X400). Panel I and II represent photomicrographs (n = 3) of the periportal and perivenular zones, respectively, in **(A)** sham group showing liver with average portal tracts, average CV, and average hepatocytes; **(B)** HIRI showing average portal tracts, and markedly necrotic hepatocytes, and others with markedly pyknotic nuclei; **(C)** OCT showing average CV with markedly congested blood sinusoids and scattered hepatocytes with pyknotic nuclei in peri-venular area; **(D)** DEF showing average portal tracts, average PV and scattered apoptotic hepatocytes in peri-portal area; **(E)** OCT and DEF showing average portal tracts, average PV and average hepatocytes. Black arrow, portal tracts; blue arrow, inflammatory infiltrate; DEF, deferoxamine; green arrow, blood sinusoids; HIRI, hepatic ischemia/reperfusion injury; OCT, octreotide; PV, portal vein; red arrow, hepatocytes; yellow arrow, hepatocytes with pyknotic nuclei.

Histological findings corroborated the improved liver function parameters. As shown in Table 2 and Figure 1, the photomicrographs of the sham group (Figure 1A) showed average portal tract, portal vein, and hepatocytes in the peri-portal zone with also average central vein, blood sinusoids, and hepatocytes in the peri-venular region. In the HIRI group (Figure 1B), the peri-portal zone exhibited mild inflammatory infiltration with a marked edematous portal tract and marked necrotic hepatocytes with some others having markedly pyknotic nuclei in both, peri-portal and peri-venular zones. Meanwhile, mildly congested blood sinusoids and areas of hemorrhage were observed in the peri-venular region. The photomicrographs of the OCT group (Figure 1C) revealed liver tissue exhibiting average portal tract, portal and central vein, with no edema or inflammatory infiltration noticed. Meanwhile, marked necrotic hepatocytes with some pyknotic nuclei in the peri-portal zone were observed. In contrast, markedly congested blood sinusoids and scattered hepatocytes with some pyknotic nuclei were shown in the peri-venular zone. In the DEF group (Figure 1D), the liver displayed average portal tract, portal and central veins, and average hepatocytes with mildly congested blood sinusoids in the peri-venular zone, in addition to scattered apoptotic hepatocytes in the peri-portal zone. No edema or inflammatory infiltration was found in both zones. Ultimately, the combinatorial group (Figure 1E) demonstrated average portal tract, blood sinusoids, and hepatocytes without edema or inflammatory infiltration in both, peri-portal and peri-venular zones.

**TABLE 2:** Zone-specific histopathological alterations in hepatic ischemia–reperfusion injury and treatment response.

Groups	Portal				Peri-venular zone			
	Portal vein	Inflammatory infiltrate	Edema	Hepatocytes	Central vein	Blood sinusoids	Hepatocytes	Inflammatory infiltrate
Control	0	0	0	0	0	0	0	0
HIRI	0	+	++	++	0	+	++	0
HIRI + Def	0	0	0	+	0	+	0	0
HIRI + Oct	0	0	0	++	0	++	++	0
HIRI + Oct/Def	0	0	0	0	++	0	0	0

For portal vein, central vein & blood sinusoids (0) = average, (+) = mildly dilated/congested, (++) = markedly dilated/congested; Inflammatory infiltrate (0) = no, (+) = mild portal; Edema (0) = no, (++) = moderate/marked; hepatocytes (0) = average, (+) = scattered apoptosis, (++) = marked apoptosis/areas of necrosis.

### Effect of OCT and/or DEF on hepatic liver enzymes and oxidative balance against hepatic ischemia/reperfusion injury in rats

**FIGURE 2:**
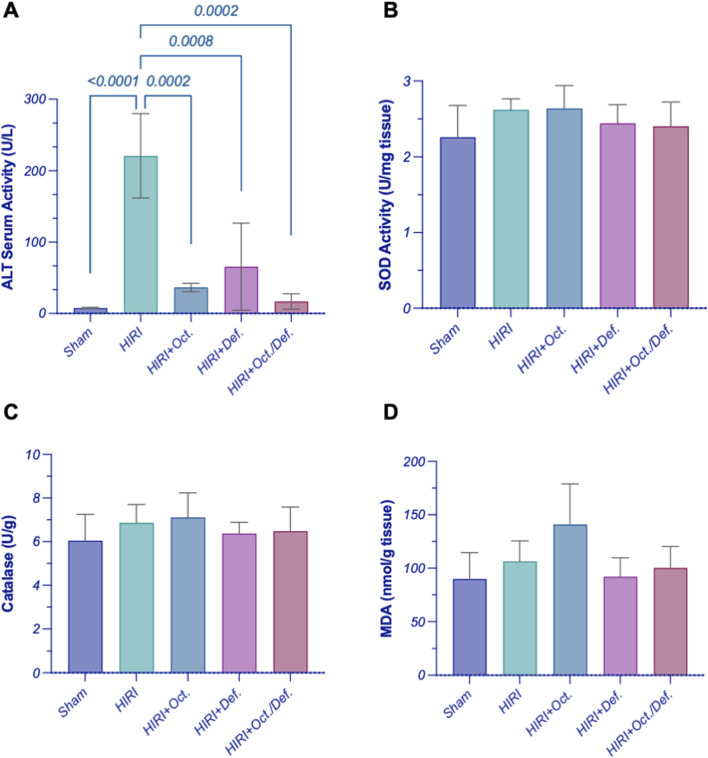
Effect of OCT and/or DEF pretreatment on the serum level of **(A)** ALT and the hepatic activity of **(B)** SOD, **(C)** CAT, and **(D)** MDA level against hepatic ischemia/reperfusion injury in rats. Values are expressed as mean ± SEM (n = 4–5). ALT, alanine aminotransferase; CAT, catalase; DEF, deferoxamine; HIRI, hepatic ischemia/reperfusion injury; MDA, malondialdehyde; OCT, octreotide; SOD, superoxide dismutase.

As depicted in Figure 2, the rats subjected to HIRI exhibited a significant elevation in the serum level of ALT by 28.7-fold (p < 0.0001), compared to the sham group. Conversely, the pretreatment with OCT, DEF, and their combination dramatically plummeted their levels by 83.5%, 70.4%, and 92.4%, relative to the HIRI group. Interestingly, the combination group resulted in a notable reduction in ALT levels, compared to each drug alone (Figure 2A). However, there were no statistically significant changes observed in the activity of both SOD and CAT, antioxidant markers, and MDA, a marker of lipid peroxidation, throughout all experimental groups (Figures 2B–D).

### Effect of OCT and/or DEF on ferroptosis markers against hepatic ischemia/reperfusion injury in rats

**FIGURE 3:**
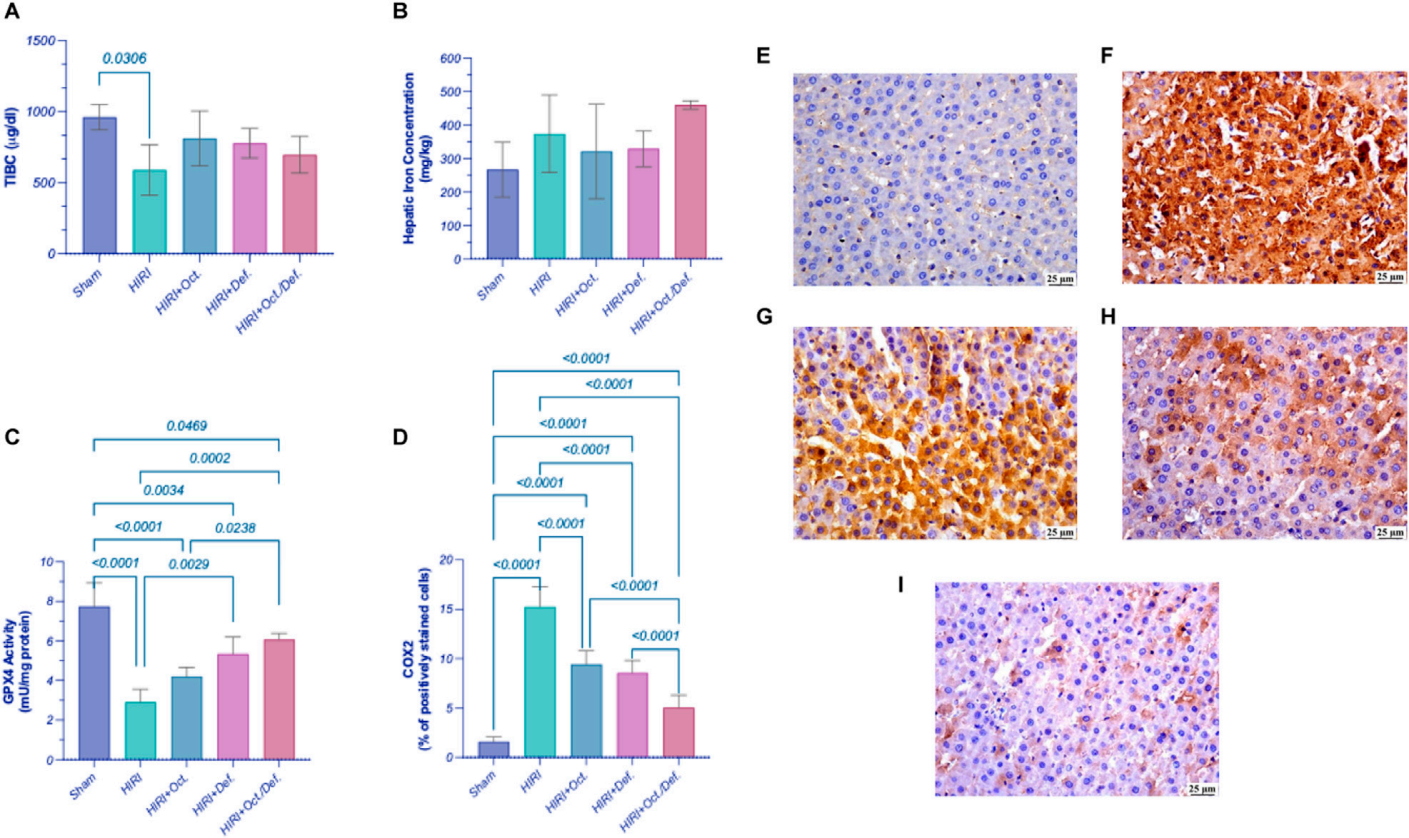
Effect of OCT and/or DEF pretreatment on ferroptosis markers against hepatic ischemia/reperfusion injury in rats. Graphs shown represent **(A)** TIBC, **(B)** hepatic iron content, **(C)** GPX4, **(D)** COX-2, panels **(E–I)** show the photomicrographs of **(E)** sham group, **(F)** HIRI group, **(G)** HIRI + OCT, **(H)** HIRI + DEF, **(I)** HIRI + OCT/DEF. Values are expressed as mean ± SEM (n = 4-5). The means of groups were calculated in **(D)** using Olympus cellSens Dimension imaging software. COX-2, cyclo-oxygenase-2; DEF, deferoxamine; GPX4, glutathione peroxidase; HIRI, hepatic ischemia/reperfusion injury; OCT, octreotide; TIBC, total iron binding capacity.

As shown in Figure 3, on one hand, the HIRI group showed a significant decrease in the concentration of TIBC by 38.6-fold (p = 0.03), compared to sham group. Although all treatment groups exhibited an increase in the concentration of TIBC, no statistically significant differences were observed either among the treatment groups or between the treatment groups and the HIRI group. On the other hand, there were no statistically significant changes observed in the concentration of the hepatic content of iron, throughout all experimental groups (Figures 3A,B).

Significantly, the HIRI group exhibited a sharp decline in the activity of GPX4, a lipid peroxidase and a key regulatory factor of ferroptosis, by 62.6% (p < 0.0001) in comparison to the sham group. In contrast, treatment with DEF and the combined therapy significantly enhanced GPX4 activity, with increases of 1.8-fold (p = 0.0029) and 2.1-fold (p = 0.0002), respectively, relative to the HIRI group. Although the OCT group displayed a 1.5-fold increase in GPX4 activity, this change did not reach statistical significance when compared to the HIRI group (Figure 3C). Furthermore, the HIRI group elucidated a considerable rise in COX-2 expression, as evidenced by its positive immuno-expression relative to the sham group. Nevertheless, the preceding administration of OCT, DEF, and the combinatorial regimen markedly displayed negative immune expression of COX-2 versus the HIRI group. Ultimately, the combination group demonstrated enhanced efficacy compared to the individual drugs administered separately as shown in the chart representing Cox-2 expression (Figure 3D). The immunohistostaining shown in the panels (Figures 3E–I) represents a normal to negative Cox-2 expression in the sham group (Figure 3E)while the photomicrograph of HIRI showed intense COX-2 expression (Figure 3F). However, OCT and DEF groups showed moderate COX-2 expression (Figures 3G,H). Notably, the photomicrograph of OCT and DEF when combined showed mild Cox-2 expression (Figure 3I).

### Effect of OCT and/or DEF on TLR4/NF-κB hub against hepatic ischemia/reperfusion injury in rats

**FIGURE 4:**
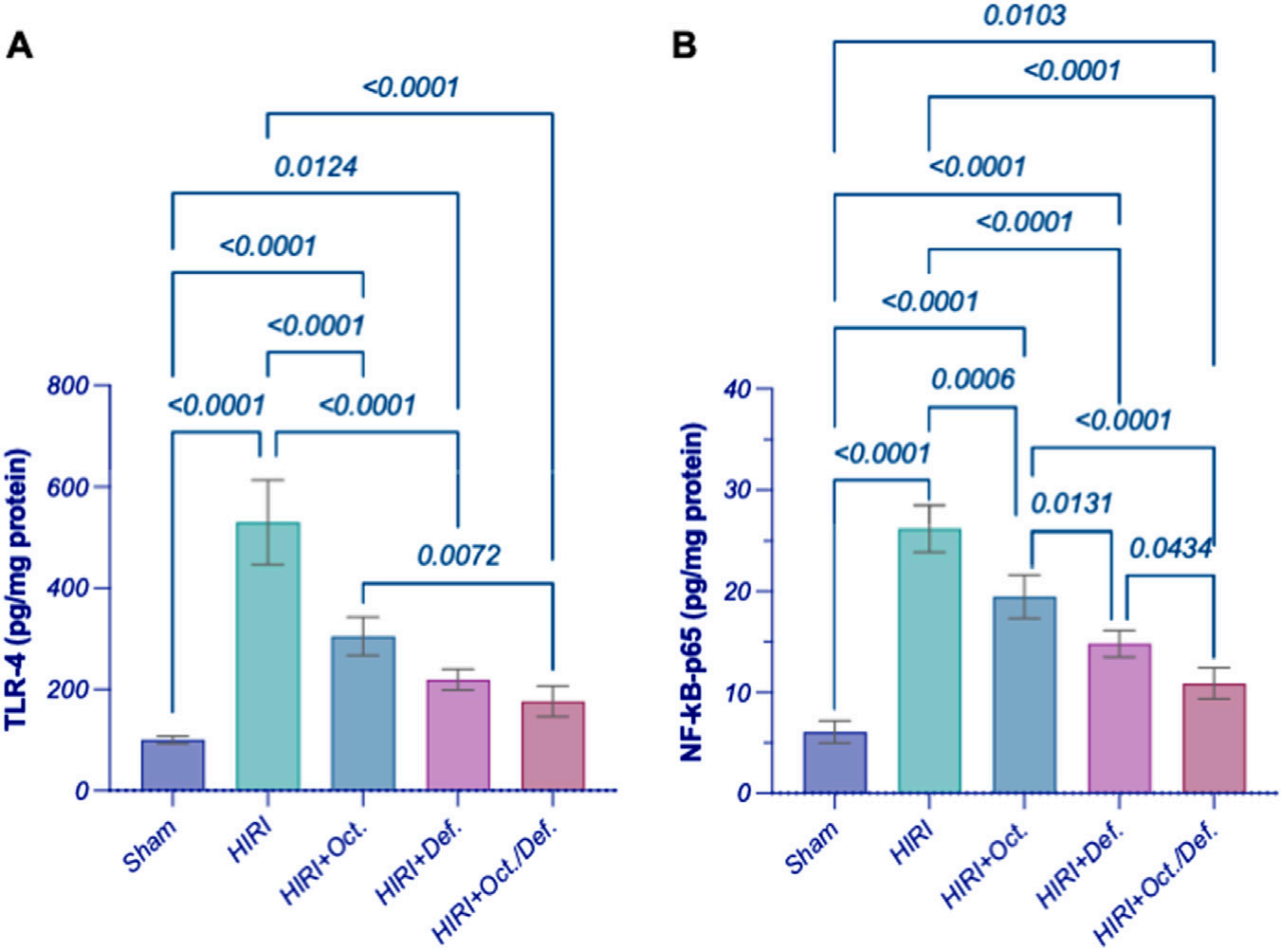
Effect of OCT and/or DEF pretreatment on the hepatic contents of **(A)** TLR4 and **(B)** NF-κB p65 against hepatic ischemia/reperfusion injury in rats. Values are expressed as mean ± SEM (n = 4–5). DEF, deferoxamine; HIRI, hepatic ischemia/reperfusion injury; NF-κB, nuclear factor kappa B; OCT, octreotide; TLR4, toll-like receptor 4.

In comparison to the sham group, the HIRI group abruptly increased the hepatic content of TLR4 by 5.3 folds, which consequently led to a 4.3-fold activation of NF-κB-p65 by (p < 0.001). In contrast, the pre-administration of OCT, DEF, and their combination significantly reversed these effects by 42.6%, 58.6%, and 66.7%, respectively (p < 0.0001) for TLR4 and 25.6, 43.5% (p = 0.006), 43.5%, and 58.4% (p < 0.0001) for NF-κB-p65, respectively, versus the HIRI group. Of note, the combined regimen had significantly lower values for the measured parameters compared to each drug alone, signifying its anti-inflammatory effects (Figure 4).

### Effect of OCT and/or DEF on inflammasome moieties against hepatic ischemia/reperfusion injury in rats

**FIGURE 5:**
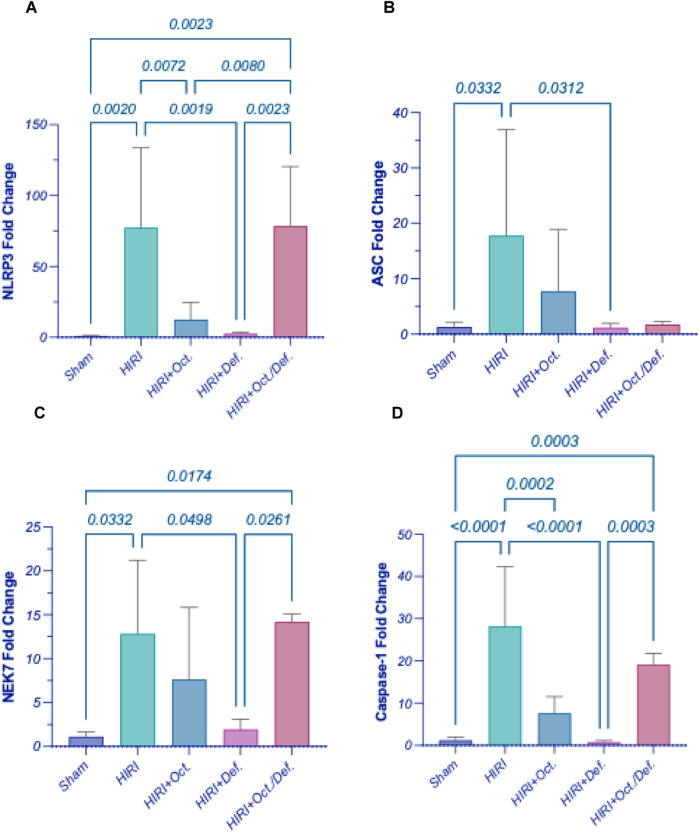
Effect of OCT and/or DEF pretreatment on the hepatic gene expressions of **(A)** NLRP3, **(B)** ASC, **(C)** NEK7, **(D)** Caspase-1 against hepatic ischemia/reperfusion injury in rats. Values are expressed as mean ± SEM (n = 4–5). ASC, apoptosis-associated speck-like protein containing a CARD; DEF, deferoxamine; HIRI, hepatic ischemia/reperfusion injury; NEK7, NIMA-related kinase 7; NLRP3, nucleotide-binding domain, leucine-rich–containing family, pyrin domain–containing-3; OCT, octreotide.

As demonstrated in Figure 5, the HIRI instigated the inflammasome hinge, as evidenced by the marked upregulation of gene expressions of NLRP3 (Figure 5A) by 8.4 fold (p < 0.0001), ASC (Figure 5B) by 13.8 fold (p = 0.0332), NEK7 (Figure 5C), a critical component in the NLRP3 inflammasome, by 11.5 fold (p = 0.0332), and caspase-1 (Figure 5D) by 23.7 fold (p < 0.0001), when compared to the sham group. Contrary to the HIRI group, the rats pretreated with DEF successfully restored all inflammasome components to their baseline levels, attaining reductions of 81.7% (p < 0.0001), 93.6% (p = 0.0312), 84.9% (p = 0.0498), and 97.3% (p < 0.0001), respectively. Furthermore, the pretreatment with OCT significantly diminished the gene expressions of NLRP3 by 57% (p = 0.0024) and Caspase-1 by 72.5% (p = 0.0002); however, it reduced the patterns of ASC by 56.5% and NEK7 by 40.9%, without achieving statistical significance, compared to the HIRI group. Significantly, no discernible alterations were observed between the combination treatment regimen and the HIRI group concerning these inflammasome markers.

### Effect of OCT and/or DEF on pyroptosis against hepatic ischemia/reperfusion injury in rats

**FIGURE 6:**
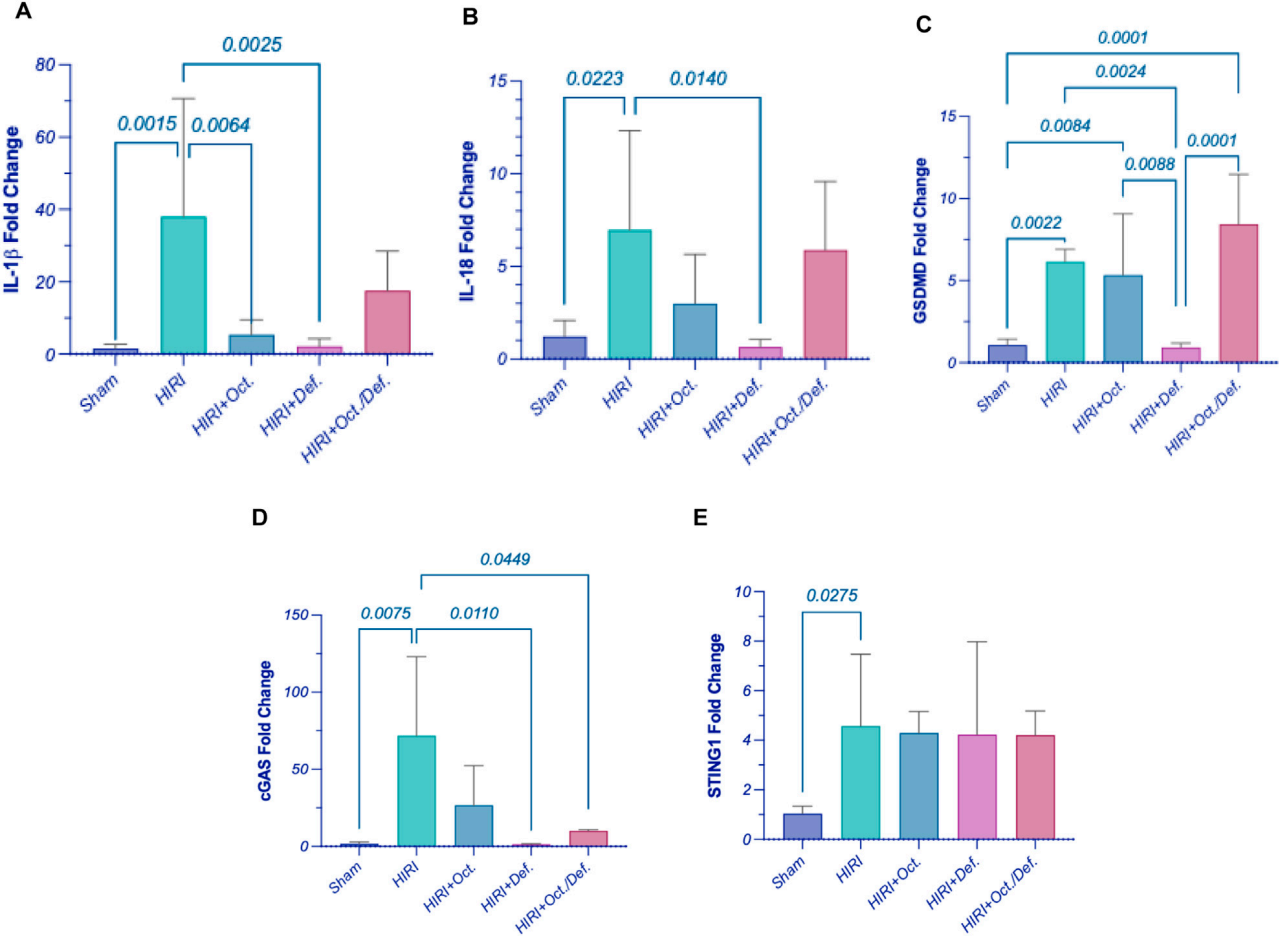
Effect of OCT and/or DEF pretreatment on the hepatic gene expressions of **(A)** IL-1β, **(B)** IL-18, **(C)** GSDMD, **(D)** cGAS, and **(E)** STING1 against hepatic ischemia/reperfusion injury in rats. Values are expressed as mean ± SEM (n = 4–5). cGAS, cyclic GMP-AMP synthase; DEF, deferoxamine; GSDMD, gasdermin D; HIRI, hepatic ischemia/reperfusion injury; IL-1β, interleukin-1 beta; IL-18, interleukin-18; OCT, octreotide; STING1, stimulator of interferon genes 1.

Consequent to the activation of inflammasome corpuscle, as illustrated in Figure 6, the gene expressions of IL-1β, IL-18, and GSDMD (Figures 6A–C) were boosted in HIRI group by 5 (p < 0.0001), 5.7 (p = 0.0223), and 2.4-folds (p = 0.0005), respectively, versus the sham group, referring to the induction of pyroptosis. Moreover, the prior administration of OCT led to a significant reduction in the gene expression of IL-1β by 62.9% (p = 0.0023) relative to the HIRI group. Nevertheless, it lowered the levels of IL-18 and GSDMD by 57.1% and 14.5%, respectively, without reaching statistical significance when compared to the HIRI group. On the contrary, the group receiving DEF succeeded in bringing pyroptotic markers to their normal level by 78.3% (p = 0.0002), 90.8% (p = 0.0140), and 61.7% (p = 0.0005), respectively, relative to the injured group. Again, no substantial differences were observed between the combination treatment regimen and the HIRI group concerning these markers.

### Effect of OCT and/or DEF on cGAS/STING1 cue against hepatic ischemia/reperfusion injury in rats

Figure 6 elucidated a marked elevation in the gene expressions of cyclic GMP-AMP synthase (cGAS) (p = 0.0075) and stimulator-of-interferon genes 1 (STING1) (p = 0.0275) by 44.6 and 4.4-fold, respectively, in hepatic ischemia/reperfused rats. Contrary to this, the rats pretreated with DEF and combined therapy showed a significant reduction in the level of cGAS by 98% (p = 0.0110) and 85.9% (p = 0.0449), respectively, compared to the HIRI group. Nonetheless, the OCT group demonstrated a reduction in cGAS levels by 62.8%, although this change did not reach statistical significance when compared to the HIRI group. Unexpectedly, the expressions of STING1 exhibited consistency across both the HIRI group and all treated groups, showing no significant differences among them (Figures 6D,E).

## Discussion

In the present study, pretreatment with deferoxamine (DEF) or when combined with octreotide (OCT) alleviated hepatic ischemia/reperfusion injury (HIRI) more obviously than OCT.A reduction in aminotransferase enzyme activity was pronounced in all groups. Meanwhile, OCT and DEF were both able to induce antioxidant activity through enhancing glutathione peroxidase 4 (GPX4) activity rather than superoxide dismutase (SOD), catalase (CAT), or malondialdehyde (MDA). Moreover, OCT and DEF reduced the protein expression of toll-like receptor-4 (TLR4) and phosphorylated nuclear factor kappa B (NFκB-p65) individually and when combined. Mechanistically, these effects might be attributed to the postulated suppression of ferroptosis and pyroptosis following the administration of each drug alone, with a prominent effect elucidated in DEF. However, on combining OCT and DEF, the potential effects were probably attributed to ferroptosis rather than pyroptosis, as the combined therapy was able to show higher activity of glutathione peroxidase 4 (GPX4), a ferroptosis inhibitor marker, and cyclooxygenase2 (COX2) inactivation, but failed to show a suppressive action on the expression of pyroptosis hallmark genes. These findings were likely a reflection of the downregulation of the cyclic GMP-AMP synthase (cGAS) gene expression without a direct alteration in stimulator-of-interferon genes (STING) itself.

Upon the induction of HIRI in rats, histopathological changes were evaluated compared to the sham group in different zones of the liver, namely, peri-portal (zone 1), peri-venular (zone 3), and the cells in between (zone 2), to validate the successful induction of the experimental model. These two zones are of particular importance in HIRI since zone 1 is the first to encounter oxygenated blood and inflammatory mediators, while zone 3 is intrinsically more hypoxia-prone and thus highly susceptible to oxidative stress and sinusoidal congestion. Their distinct metabolic and hemodynamic vulnerabilities explain the zonated pattern of hepatocellular injury and necrosis observed in HIRI, and evaluating both compartments is critical to capture potential zone-specific treatment effects. Hepatic injury was manifested in the HIRI group by mild inflammatory infiltration in the peri-portal zone with a marked edematous portal tract and marked necrotic hepatocytes with some others having markedly pyknotic nuclei in peri-venular as well as peri-portal zone. Meanwhile, mildly congested blood sinusoids and areas of hemorrhage were observed in the peri-venular region. These changes were mitigated modestly with OCT and prominently with DEF as the liver sections pretreated with OCT or DEF exhibited average portal tract, portal and central vein with no edema or inflammatory infiltration. Meanwhile, DEF demonstrated average hepatocytes with mildly congested blood sinusoids in the peri-venular zone with scattered hepatocytes in the pre-portal one. In contrast, OCT displayed marked necrotic hepatocytes with some pyknotic nuclei in the peri-portal zone, markedly congested blood sinusoids, and scattered hepatocytes with some pyknotic nuclei in the peri-venular zone. Collectively, the present findings suggest the therapeutic potential of OCT and DEF against HIRI with superiority to DEF over OCT. On the histopathological level, it was obvious that the combinatorial therapy was able to restore the normal architecture of hepatocytes, portal tract, and blood sinusoids in the two zones, meanwhile, it showed no inflammatory signs. This highlighted the enhanced protective effect against HIRI achieved by the combination regimen.

On the functional level, hepatic injury was verified by the exalted activity of serum alanine transaminase (ALT), a specific biochemical marker for hepatocellular injury (Peralta et al., 2013). The current findings demonstrated that pre-treatment with OCT, DEF, and their combination normalized the activity of ALT by dramatically plummeting its prominent elevation in the HIRI group. The combination regimen showed the highest decline in ALT activity among all treated groups, although it did not attain statistical significance compared to individual treatments. This finding aligns with previous reports where serum ALT level showed a nearly 15-fold increase with HIRI in the same model (XUE et al., 2016). The reductions in ALT levels observed with DEF and OCT were also in agreement with prior studies demonstrating their protective effects in HIRI (El-Sisi et al., 2021).

Mechanistically, many actions were reported to contribute to the palliation of the injury occurring after IR in the liver. Among these mechanisms is the antioxidant action which serves as a first-line defense mechanism that neutralizes the released free radicals and oxidative stress that may arise in the HIRI (George et al., 2024). In the current study, it was hypothesized that the protective effect showed on the histological and functional levels when administering OCT and/or DEF before the HIRI might be attributed to the previously reported antioxidant action of OCT and DEF, each alone (Shimoni et al., 1994; Aziz et al., 2018; Cavdar et al., 2024). Unexpectedly, the antioxidant SOD and CAT activities assessed in this study remained unchanged across all experimental groups, with no substantial differences among them. The unpredicted stable activities of the antioxidant SOD and the peroxidase CAT in the HIRI group might be attributed to either the neutralization of the oxidative stress with the antioxidant capacity of the liver during the IR process (Slater, 1984) or the implication of other alternative oxidative players in hepatic injury in the HIRI group. Therefore, oxidative stress level was further investigated by measuring MDA, a well-established marker of lipid peroxidation induced by free radicals (Slater, 1984). Similarly, no change in MDA levels among experimental groups was observed, suggesting that oxidative damage likely occurred through a similar underlying mechanism under the given experimental settings.

On the other hand, the phospholipid hydroperoxidase enzyme, GPX4, which is responsible for the reduction of the hydroperoxides of the cell membrane phospholipids into non-toxic lipid alcohol to protect the cell from the accumulation of the toxic iron-dependent lipid ROS (Forcina and Dixon, 2019), was assessed to investigate its likely implication in the postulated antioxidant capacity of the treatments. Intriguingly, GPX4 was significantly reduced in the HIRI group, whereas on pretreatment with DEF alone or combined with OCT, the notable loss in GPX4 activity following the HIRI was overturned considerably compared to the untreated group. The OCT group, however, increased GPX4 activity, yet with no significant difference vs. the HIRI group. It is worth mentioning that the combined therapy displayed superior antioxidant activity compared to OCT alone, as evidenced by the enhanced GPX4 activity.

Collectively, this might indicate that the oxidative stress in the current model of HIRI might be restricted to iron-dependent localized cell membrane phospholipid peroxidation, manifested by lowered GPX4 activity without any alteration in the SOD, CAT activities or MDA levels. The elevated GPX4 antioxidant activity upon pretreatment with the iron chelator DEF alone or when combined with OCT, contrary to OCT alone, supports a role for iron-mediated oxidative stress, pointing out a probable role for ferroptosis in this model. Ferroptosis in HIRI occurs through oxidative non-apoptotic programmed cell death potentiated by the accumulation of the intracellular labile iron pool and inhibition of GPX4, which utilizes glutathione in exerting its antioxidant action, leading to mitochondrial damage (Luo et al., 2021). Reduced GPX4 activity is known as a ferroptosis hallmark, and DEF, as an iron chelator, can suppress its ferroptotic action (Forcina and Dixon, 2019). Briefly, these findings might entail the involvement of ferroptosis as a source of oxidative stress in the HIRI, which is in line with Sun et al. (2022) and was confirmed by the previously reported ability of DEF to alleviate liver injury (Yamada et al., 2020).

To study the implication of ferroptosis in the current HIRI model, serum total iron binding capacity (TIBC) was assessed as a marker for the total capacity of transferrin, the iron plasma transport protein, to bind to ferric ions in the blood (Yamada et al., 2020; Faruqi et al., 2025). A significant reduction in the TIBC was observed in the HIRI group compared to the sham group, indicating a lower capacity of iron binding and consequently, transferrin saturation with ferric ions in the blood of HIRI untreated rats, which implies serum iron overload. This can explain the higher unbound or free iron in the HIRI compared to the sham group. Conversely, treated groups exhibited an increase in TIBC -albeit insignificant-compared to HIRI. Moreover, liver tissues were investigated for their total iron content by using the inductively coupled plasma optical emission spectrometry (ICP-OES) technique, a sensitive method for measuring trace element content, like iron, in tissue samples (De la Guardia and Armenta, 2011). The total iron content in the tissues displayed a similar level in all tested groups, including the HIRI group. This finding does not negate ferroptosis, as total iron content is not a specific indicator of ferroptotic activity, instead, labile iron (ferrous ions) is the main driver for ferroptosis (Lei et al., 2024). Given that total iron measurement includes both free labile and free iron forms, considering other specific ferroptotic indicators is of the essence to confirm ferroptosis.

Accordingly, ferroptosis was further verified using an additional marker, prostaglandin-endoperoxide synthase 2 (PTGS2), a downstream target regulated by GPX4-mediated ferroptosis, which encodes the COX-2 protein (Yang et al., 2014). An upsurge in COX-2 immunoexpression in the HIRI group was reported relative to the sham, an observation that verifies the ferroptosis incident. All treated groups demonstrated negative immunoexpression of COX-2 vs. HIRI group. The combination group, however, demonstrated superior efficacy when compared to individual treatments, confirming the modulatory action of both drugs on lipid peroxidation, as evidenced by changes in COX-2, being a downstream marker of ferroptosis-dependent lipid peroxidation (Yang et al., 2014). Collectively, the results highlighting GPX4 as a ferroptosis inhibitor and COX-2 as a downstream ferroptosis marker suggest that combining DEF with OCT might alleviate HIRI by suppressing nonapoptotic iron-dependent programmed cell death or ferroptosis through the activation of GPX4 and COX-2 signaling. Accordingly, DEF’s efficacy might stem from a targeted intervention against a specific type of oxidative damage rather than broad-spectrum antioxidant enzyme upregulation. Similarly, the observed suppression of COX-2 further supports an anti-ferroptotic mechanism, consistent with previous studies (Javali et al., 2023), suggesting that this activity might not directly influence SOD or CAT levels. Therefore, the protective effects observed in the current study likely reflect the interruption of the downstream propagation of injury through ferroptosis, rather than a global shift in canonical antioxidant enzyme activity.

Since HIRI causes the release of damage-associated molecular patterns (DAMPs) (Yamada et al., 2020), which are also released from the injured cell membrane triggered by ferroptosis, the activation of TLR4 signaling ensues (Li et al., 2019). This activation subsequently activates its downstream NFκB, whose activity can be reversibly inhibited by GPX4 (Chen et al., 2023; Li et al., 2018) towards potentiating inflammatory reactions. Therefore, to confirm the implication of this pathway given the current experimental settings, the protein levels of TLR4 and NFκB-p65 were assessed. HIRI abruptly increased the hepatic content of TLR4 and significantly activated NFκB-p65 compared to the sham group, denoting the potentiation of inflammatory response in the studied model. Interestingly, the pre-treatment with OCT and/or DEF significantly reversed these effects for TLR4 and NFκB-p65 compared to the sham group. Notably, the combined regimen significantly lowered TLR4 expression compared to OCT, while it prominently lowered NFκB-p65 compared to each drug alone, underscoring the superior anti-inflammatory activity of the combined therapy over monotherapies.

To further dissect the downstream trajectory of the inflammatory response shown in the HIRI model following the activation of TLR4/NFκB as well as ferroptosis, and considering previous insights highlighting the role of pyroptosis in HIRI and identifying OCT as a pyroptosis inhibitor (El-Sisi et al., 2021), we hypothesized that pyroptosis might be a possible target for our treatments, both individually and in combination. Thus, the gene expression of pyroptosis key players like NOD-, LRR- and pyrin domain-containing protein 3 (NLRP3) receptor or sensor, the adaptor domain apoptosis-associated speck-like protein containing a CARD (ASC), caspase-1, NIMA-related kinase 7 (NEK7), interleukin (IL)-1β, IL-18 and Gasdermin D (GSDMD), were investigated. Consequent to the activation of inflammasome corpuscle in the HIRI group, the gene expression of IL-1β, IL-18, and GSDMD was upregulated compared to the sham group, implying pyroptosis induction. Moreover, pre-treatment with OCT led to a significant reduction in the gene expression of IL-1β relative to the HIRI group. Nevertheless, it lowered the levels of IL-18 and GSDMD; however these reductions were not statistically significant compared to the HIRI group. On the contrary, the group receiving DEF succeeded in bringing pyroptotic markers to their normal level. The current findings interestingly highlighted the ability of DEF to inhibit pyroptosis through suppressing IL-1β, IL-18, and GSDMD following its suppression to the inflammasome complex more prominently than OCT. Surprisingly, the combined regimen failed to repress pyroptosis despite each drug demonstrating anti-pyroptotic activity individually. Collectively, this might imply that the combination of the two drugs probably had no additive effect on the mRNA level in the current model. Accordingly, the observed anti-inflammatory activity of the combined regimen likely stems predominantly from its anti-ferroptotic activity, inhibition of TLR4/NFκB, in addition to the activation of GPX4/COX2 cue rather than pyroptosis. However, this apparent lack of suppression of pyroptotic genes might not necessarily indicate antagonism but rather highlights the complexity of the inflammatory landscape, where not all molecular pathways may align uniformly with phenotypic outcomes.

Recent reports have proved that inflammatory pathways affect ferroptosis, but the precise molecular mechanisms are still debatable. Pyroptosis, TLR4/NFκB, and ferroptosis were believed to be interrelated together with cGAS/STING1 pathway, which is activated by mitochondrial DNA leakage of injured cells (Chen et al., 2023). Another study indicated that elevated intracellular iron overload, not only could potentiate ferroptosis, but might also activate NLRP3 inflammasomes, inducing pyroptosis through the activation of cGAS/STING pathway (Gupta et al., 2023). Consequently, the current work aimed at studying the role of cGAS/STING1 gene expression on pyroptosis and ferroptosis cues in the current model. A marked elevation in the gene expressions of cGAS and STING1 in hepatic ischemia/reperfused rats was noticeable. Contrariwise, rats pretreated with DEF, alone or combined with OCT, showed a significant reduction in the expression of cGAS mRNA compared to the HIRI group. Nonetheless, the OCT group demonstrated a reduction in cGAS mRNA expression despite failing to achieve statistical significance when compared to the HIRI group. Unexpectedly, the expressions of STING1 mRNA exhibited consistency across both the HIRI group and all treated groups, showing no significant differences among them. Therefore, the positive relation between cGAS/STING1 and ferroptosis in the current model was obvious, which is in line with the reported activity of cGAS/STING1 as a ferroptosis promoter (Li C. et al., 2021; Ding et al., 2024). However, the ability of DEF alone or when combined with OCT to reduce the gene expression of cGAS without altering STING1 mRNA. These downregulations in cGAS gene expression might suggest a reduction in the initial sensing of DNA damage or inflammatory signals, which aligns with the overall anti-inflammatory and protective effects seen with the treatments. However, the unchanged STING1 mRNA expression implied that the treatments specifically modulated the expression of the cGAS sensor, rather than broadly altering the entire cGAS/STING1 pathway’s scaffolding protein. Probably, the studied interventions appeared to target the upstream cGAS component specifically, directly inhibiting cGAS mRNA expression, reducing the presence of its DNA ligands, or preventing cGAS upregulation that typically occurred during HIRI (Hopfner and Hornung, 2020). While STING1 is crucial for many cGAS-driven responses, its unchanged mRNA expression advocated that the observed benefits were primarily driven by cGAS mRNA supression; meanwhile, other pathways might be compensating for STING1’s role. This finding could point to an initial inflammatory trigger (cGAS) without affecting the broader immune signaling platform (STING1), which might have fewer off-target effects (Holm et al., 2016; Samson and Ablasser, 2022).

**FIGURE 7:**
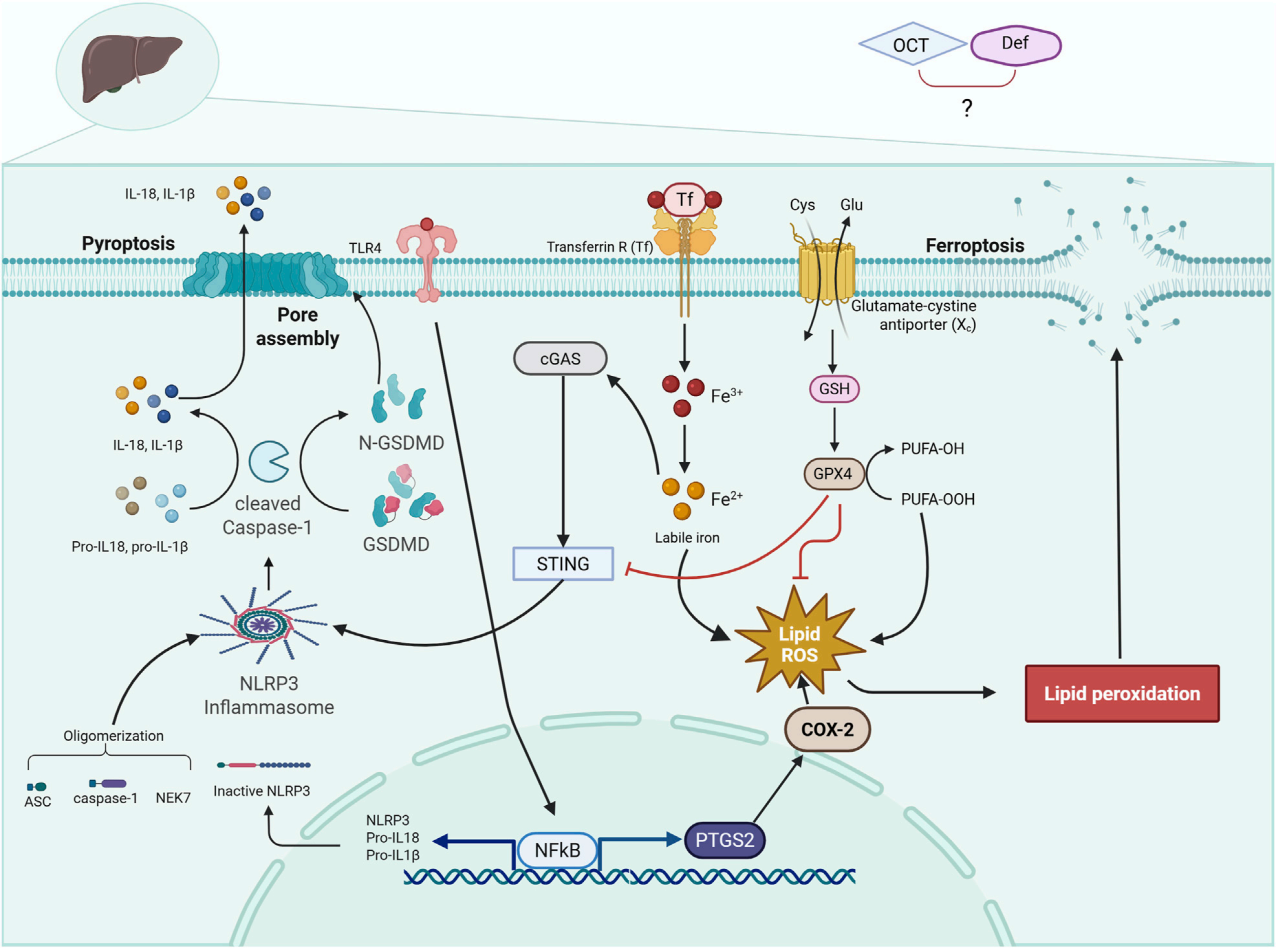
Diagrammatic representation of the cross-talk between ferroptosis and pyroptosis trajectories. The diagram illustrates a crosstalk between two programmed cell death, ferroptosis and pyroptosis, in HIRI, where the activation of TLR4/NF-κB and NLRP3 inflammasome leads to pyroptosis. Meanwhile, HIRI causes an accumulation of iron and PUFA peroxidation, depleting GSH and GPX4, leading to ferroptosis. ASC, apoptosis-associated speck-like protein containing a CARD; cGAS, cyclic GMP-AMP synthase; COX-2, cyclo-oxygenase-2; DEF, deferoxamine; GPX4, glutathione peroxidase 4; GSDMD, gasdermin D; GSH, glutathione; HIRI, hepatic ischemia/reperfusion injury; IL-1β, interleukin-1 beta; IL-18, interleukin-18; NEK7, NIMA-related kinase 7; NF-κB, nuclear factor kappa B; N-GSDMD, N-terminal gasdermin D; NLRP3, NOD-like receptor protein 3; OCT, octreotide; PTGS2, prostaglandin-endoperoxide synthase 2; PUFA, polyunsaturated fatty acids; STING1, stimulator of interferon genes 1; TF, transferrin; Transferrin R, transferrin receptor; TLR4, toll-like receptor 4. Solid arrows indicate activation, and flat-headed lines indicate inhibition.

Taken together, and as illustrated in Figure 7, the present findings demonstrate that OCT and DEF, alone or combined, might alleviate HIRI on both structural and functional levels. This protective effect is primarily attributed to the antioxidant and lipid-peroxide scavenging activity of GPX4, rather than by classical antioxidant enzymes such as SOD or CAT or by the non-enzymatic oxidative stress marker MDA, resulting in probable suppression of ferroptosis, as manifested by COX-2 downregulation as well. DEF depicted a more pronounced protective effect to alleviate HIRI in rodents compared to OCT, an outcome likely attributable to its obvious antioxidant and anti-inflammatory activities, which converge on ferroptosis suppression via GPX4 activation and paralleled inactivation of COX-2, TLR4/NFκB, cGAS mRNA expression, and pyroptosis. Combinatorial therapy revealed more pronounced effects compared to monotherapy in GPX4, COX-2, and TLR4/NFκB axis, although it exhibited an absence of an additive effect in the regulation of pyroptosis. To further optimize therapeutic strategies and fully clarify these complex interactions, particularly the unanticipated nonadditive antipyroptotic effect with combined therapy, future studies can explore alternative dosing regimens and temporal sequencing of OCT and DEF. Validation of these findings under more severe HIRI paradigms, such as extending ischemia to 60–90 min or reperfusion to 6–24 h, in another animal strain might provide insights into durability and broader translational potential.

Although the present work is exploratory and mechanistically focused on an *in vivo* HIRI model, it also highlights limitations that shape future directions. Measuring aspartate transaminase (AST) can reinforce the results of ALT to confirm the modulatory effects of the tested drugs on HIRI. Moreover, mechanistic confirmation could be strengthened by complementary assays, including BODIPY- C11 lipid ROS quantification, assessment of 4-HNE, ACSL4 expression, and labile iron pools or transferrin levels. In addition, multi-modal analysis of the TLR4/NFκB pathway and related inflammatory mediators, protein levels of cleaved caspase-1 p20, GSDMD-N, or IL-1β/IL-18 maturation, serum lactate dehydrogenase (LDH), and the use of genetic knockout models or selective inhibitors targeting the implicated signaling axes could rigorously confirm the current findings. Meanwhile, assessing pyroptosis in a later reperfusion time point (e.g., 6–24 h) rather than a single timepoint (3 h) may help in capturing the changes in pyroptotic gene expression. From a translational perspective, both OCT and DEF hold promise owing to their established safety profiles and clinical use in other indications. While direct studies in HIRI remain lacking, future investigations employing *ex vivo* human tissue models or organoid systems will be critical to bridge the experimental-clinical gap and validate their therapeutic potential. Ultimately, translation of these insights into clinical contexts will require rigorously designed trials in liver transplantation or hepatic surgery cohorts, where the feasibility and efficacy of OCT and DEF (individually or in combination) as adjunctive strategies to mitigate ischemia-reperfusion injury can be systematically evaluated.

## Data Availability

The original contributions presented in the study are included in the article/supplementary material, further inquiries can be directed to the corresponding author.
